# Unequal impact of the COVID-19 crisis on minority ethnic groups: a framework for understanding and addressing inequalities

**DOI:** 10.1136/jech-2020-216061

**Published:** 2021-04-21

**Authors:** Srinivasa Vittal Katikireddi, Sham Lal, Enitan D Carrol, Claire L Niedzwiedz, Kamlesh Khunti, Ruth Dundas, Finn Diderichsen, Ben Barr

**Affiliations:** 1 MRC/CSO Social and Public Health Sciences Unit, University of Glasgow, Glasgow, UK; 2 Department of Disease Control, London School of Hygiene and Tropical Medicine, London, UK; 3 Institute of Infection, Veterinary and Ecological Sciences, University of Liverpool, Liverpool, UK; 4 Institute of Health & Wellbeing, University of Glasgow, Glasgow, UK; 5 Primary Care and Public Health, Imperial College London, London, UK; 6 Department of Public Health, University of Copenhagen Faculty of Health and Medical Sciences, Copenhagen K, Denmark; 7 Public Health and Policy, University of Liverpool, Liverpool, UK

**Keywords:** ethnicity, health inequalities, COVID-19, social epidemiology

## Abstract

Minority ethnic groups have been disproportionately affected by the COVID-19 pandemic. While the exact reasons for this remain unclear, they are likely due to a complex interplay of factors rather than a single cause. Reducing these inequalities requires a greater understanding of the causes. Research to date, however, has been hampered by a lack of theoretical understanding of the meaning of ‘ethnicity’ (or race) and the potential pathways leading to inequalities. In particular, quantitative analyses have often adjusted away the pathways through which inequalities actually arise (ie, mediators for the effect of interest), leading to the effects of social processes, and particularly structural racism, becoming hidden. In this paper, we describe a framework for understanding the pathways that have generated ethnic (and racial) inequalities in COVID-19. We suggest that differences in health outcomes due to the pandemic could arise through six pathways: (1) differential exposure to the virus; (2) differential vulnerability to infection/disease; (3) differential health consequences of the disease; (4) differential social consequences of the disease; (5) differential effectiveness of pandemic control measures and (6) differential adverse consequences of control measures. Current research provides only a partial understanding of some of these pathways. Future research and action will require a clearer understanding of the multiple dimensions of ethnicity and an appreciation of the complex interplay of social and biological pathways through which ethnic inequalities arise. Our framework highlights the gaps in the current evidence and pathways that need further investigation in research that aims to address these inequalities.

## Introduction

Minority ethnic groups have been disproportionately affected by the COVID-19 pandemic, with the clearest evidence from the UK and the USA.^1–4^ While the exact reasons for this remain unclear, they are likely due to a complex interplay of a number of factors rather than a single cause. Reducing these inequalities requires a greater understanding of the causes. Research to date, however, has been hampered by a lack of theoretical understanding of the meaning of ‘ethnicity’ or the potential pathways leading to ethnic inequalities.

In this paper, we describe a framework for understanding the pathways that have generated ethnic inequalities in COVID-19—to our knowledge, the first of its kind. Current research provides only a partial understanding of some of these pathways. Future research and action will require a clearer understanding of the complex dimensions of ethnicity and an appreciation of the complex interplay of social and biological pathways through which ethnic inequalities arise. Our framework highlights the gaps in the current evidence and pathways that need further investigation in research that aims to address these inequalities.

## Understanding ethnicity

Ethnicity is socially constructed.^5^ It can be defined as a ‘social group a person belongs to, and either identifies with or is identified with by others, as a result of a mix of cultural and other factors including language, diet, religion, ancestry, and physical features traditionally associated with race’.^6^ Ethnicity is therefore a complex concept which includes multiple dimensions including country of birth, language, religion and culture. Although it is socially constructed, it may be associated with biological attributes such as skin colour, that influence the unequal treatment of people within racist societies. The act of categorising people into ethnic groups is a social process, influenced by particular social, cultural and historical contexts. For this reason, ethnic categories differ across the world, with the same term often referring to different groups of people—for example, the term ‘Asian’ is often understood as referring mainly to East Asian people in the USA whereas in the UK the same term is typically interpreted as including people from the Indian subcontinent.^5^

In this paper, we therefore use the term ethnicity throughout but include the concept of race within this term (as defined above) and consider racial inequalities as core to ethnic inequalities. This reflects a tradition in the UK of focusing on ethnic inequalities in health, but we acknowledge inter-related dimensions on inequality are often given greater emphasis in different countries. For example, in the USA the term race is more widely used, with the socially constructed nature of racial categories also explicitly acknowledged by public health researchers.^7^ Similarly, in many European countries outside of the UK the health of migrants (classified on the basis of country of birth) has often been the focus of research, rather than minority ethnic groups—at least in part due to a lack of data collection on ethnicity. While we use terminology related to ethnicity throughout the remainder of the paper, we believe our framework and the arguments expressed broadly apply to inequalities related to migrant status and similar related inequalities. The use of ethnicity also allows us to include inequalities experienced within broader racial groups—for example, by white traveller and gypsy communities across Europe.^8^ We also note that ethnic groups that experience disadvantage can be numerical majorities in some countries and our use of the term minority ethnic also refers to relative power within society.^9^

While not all minority ethnic groups in all countries experience worse health than the majority ethnic group,^10^ differences in health across ethnic groups, in terms of both morbidity and mortality, have been repeatedly documented in the UK and other countries.^11^ It is important to note that the health and related experiences of minority ethnic groups are not homogenous, with different patterns seen depending on which health outcomes are studied.^5^ While understanding by current researchers has largely moved on from racist scientific thinking of the 19th century that narrowly viewed these differences through a biological lens,^7^ this is not universally the case.^12^ The multiple dimensions of ethnicity influence health through their interaction with wider social processes. In the past, social disadvantage, and particularly experiences of racism, have been downplayed as explanations for these differences. However, there is now increasing recognition of the role of structural racism. Processes of racialisation are contingent on socio-historical context, such that some groups may be more or less racialised at different times. For example, white Irish people living in the UK were subject to substantial and overt racism in the early 20th century, with other racial groups (such as white Eastern European and travelling community groups) being more targeted at the end of the century.^13^

Structural racism has been defined as ‘the macrolevel systems, social forces, institutions, ideologies, and processes that interact with one another to generate and reinforce inequities among racial and ethnic groups’.^14^ The term draws attention to the way these ethnic inequalities arise not only from the intended actions of individuals, but rather from broader societal mechanisms. For example, historical experiences of minority ethnic groups and long-term discrimination may lead to a higher proportion working in lower paid jobs on insecure contracts without sickness benefits and in public-facing occupations, living in crowded housing conditions, and having fewer resources for health (eg, education, income).^15^ These factors are likely to increase psychosocial stress, mental health problems and harmful health behaviours (eg, smoking, poor diet and physical inactivity). There is also a wealth of evidence documenting inequalities faced by minority ethnic groups in accessing quality healthcare.^5^ Healthcare planning may not take into account different experiences, perceptions and expectations of ethnic minorities, and therefore health services may not meet the needs of some ethnic groups—further widening inequalities.^5^ Reported experiences of racial discrimination are also associated with poorer health.^16^ This includes both interpersonal racism (which refers to discriminatory actions between individuals) and institutional racism (when discriminatory policies and practices are embedded in organisations). While ethnic inequalities in health are often linked to socioeconomic differences, they are not entirely explained by these factors due to the experiences of discrimination and intersecting inequalities within and across social groups.

Studies of ethnic differences in health have not paid sufficient attention to the social processes that give rise to these inequalities. Often studies include ‘ethnic group’ as one of many variables in regression models ‘controlling or adjusting’ for clinical, social and economic factors that are in fact important explanations of ethnic inequalities. This process has resulted in researchers sometimes erroneously concluding that ethnic inequalities do not exist (eg, see Yehia and colleagues^17^). Adjusting away the pathways through which inequalities actually arise (ie, mediators) can lead to the effects of social processes, and particularly structural racism, becoming hidden. This ‘black box epidemiology’, which has been often used in recent studies of ethnic inequalities in COVID-19,^18^ has been rightly criticised for ignoring the theory underpinning analyses.^19^ More theory-informed analyses can help yield more informative insights.

## Framework to understand ethnic inequalities in the health impacts of the pandemic

**Figure 1:**
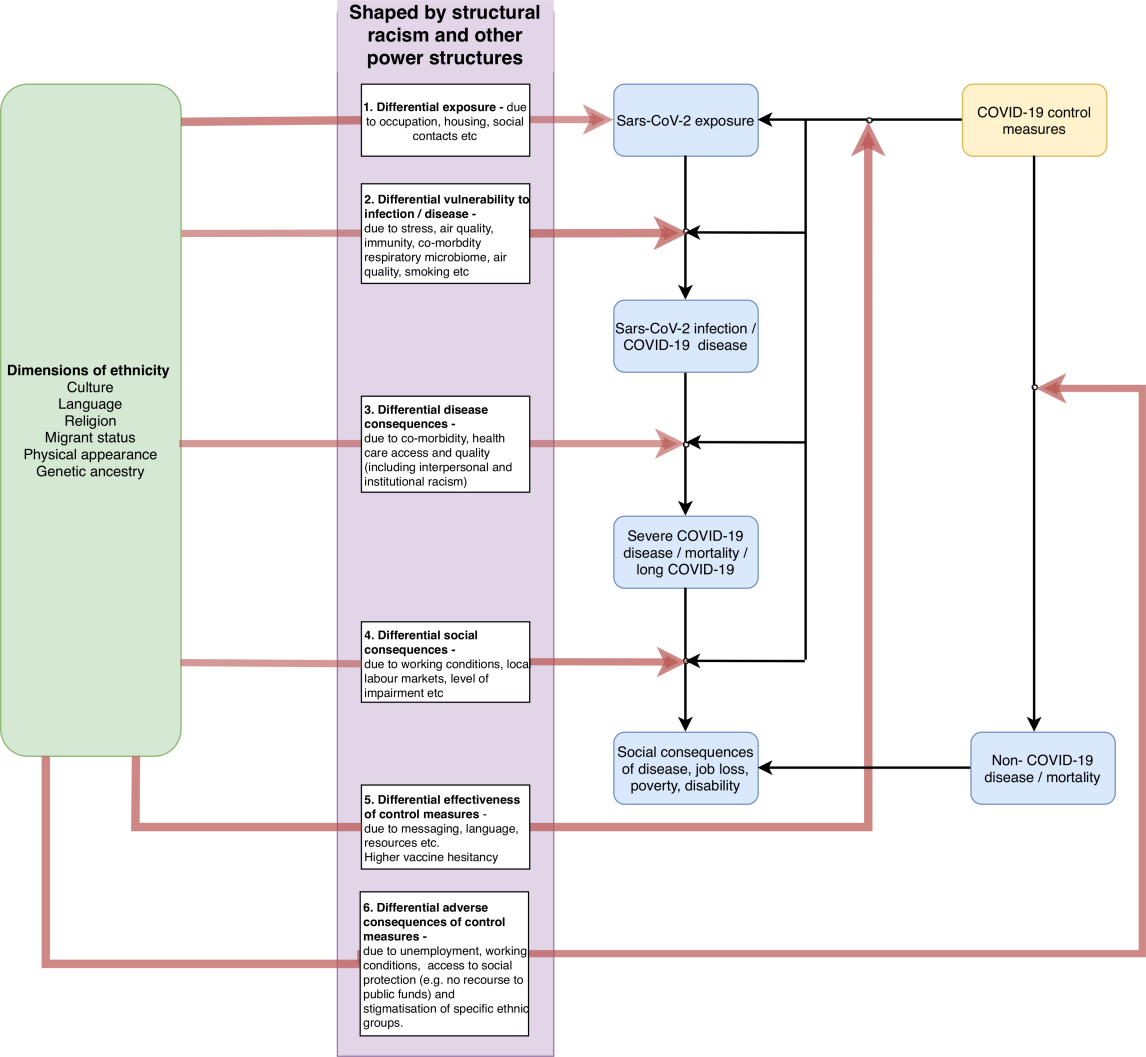
A framework for understanding pathways underpinning ethnic inequalities in COVID-19 and potential targets for policy.

To inform analyses of ethnic inequalities in the health impacts of the COVID-19 crisis, we present a framework of the potential mechanisms and pathways that could contribute to health differences between ethnic groups (figure 1). We build on a well-established framework for studying health inequalities^20 21^ which distinguished the individual proximate causes of disease from their societal causes and highlighted the potential importance of differential exposure to causes of disease, differential vulnerability to their effects and differential consequences of disease. In our model, we suggest that differences in health outcomes due to COVID-19 could occur at multiple stages: from exposure to the virus, development of disease, and through the indirect impacts of control measures and management of an individual with COVID-19. At each step, ethnic inequalities could develop through social and economic mechanisms which have biological effects. A comprehensive understanding of these pathways will help identify targets for policy interventions, as well as future research. We provide a brief introduction to each element of the framework, drawing on relevant studies to illustrate how it might be relevant to ethnic inequalities in health arising from the pandemic. We note that we have not conducted a systematic assessment of the evidence base in relation to each of these pathways and we therefore provide these studies for illustrative purposes only.

### Differential exposure

Minority ethnic groups could experience greater exposure to the virus and therefore higher risk of infection, which could relate to the frequency of contact or the potential infective dose of each contact. For example, working in specific occupations (eg, health and social care workers, transport workers) or living in overcrowded housing could lead to being in contact with potentially infected persons more frequently and for a longer duration potentially leading to a higher viral load.^22 23^ Recent findings from a representative English infection survey found evidence that all minority ethnic groups studied were more likely to have serological evidence of previous SARS-CoV-2 infection compared with the majority white British population.^24^

### Differential vulnerability to infection/disease

Minority ethnic groups could be more likely to develop disease once exposed. This could result from differences in nutritional status, comorbidities and immune response, which themselves could be driven by stress or environmental conditions, such as air pollution. There is considerable evidence that incidence of clinically important disease (reflected by positive symptomatic tests or hospitalisations) are higher in many minority ethnic groups.^2 18^ However, it is not clear if this merely reflects greater exposure or differences in vulnerability to developing disease. To differentiate vulnerability to the disease from greater exposure, analysis would need to compare ethnic differences in the risk of reporting symptomatic disease among both symptomatic and asymptomatically infected people.

### Differential disease consequences

Of those with disease, some minority ethnic groups may be more likely to develop severe disease, require mechanical ventilation, experience complications and potentially die. This could, for example, be due to differences in underlying comorbidities or differential access to healthcare between ethnic groups.^23^ A large cohort study of nearly 35 000 UK hospitalisations found a 30% increased relative risk of critical care admission and mechanical ventilation among people from South Asian, black or minority ethnic groups and this relationship was still present after adjusting for age and sex.^25^ After accounting for some potential explanations of this increased risk (such as comorbidities like diabetes), minority ethnic groups were still more likely to require critical care and mechanical ventilation than white groups. Different ethnic groups may be at risk of longer term health consequences, such as greater risks of ‘long COVID-19’ (also referred to as post-COVID-19 syndrome),^26^ but evidence is currently limited.

### Differential social consequences

Minority ethnic groups may also experience differential social consequences following recovery from COVID-19 disease. COVID-19 disease may lead to long-lasting disability that results in job loss and future loss of earnings due to poor health. One important reason for a potential disproportionate impact on minority ethnic groups is the often higher levels of insecure employment (such as self-employment or being on ‘zero hours’ contracts) that were already experienced prepandemic. Contracting COVID-19 disease, and especially ‘long COVID-19’, could lead to minority ethnic people being more likely to experience job loss and poverty. However, empirical data remain relatively scarce on the social consequences of the disease^26^ and further research is required to address this gap.

### Differential effectiveness of control measures

Public health interventions designed to control the pandemic may also, in themselves generate ethnic health inequalities, as their impact on risk of exposure, vulnerability and consequences (pathways 1–4) may be different for some ethnic groups, sometimes also referred to as intervention-generated inequalities.^27^ We believe conceptualising of the differential effectiveness of control measures as a distinct pathway is important given the necessity of understanding the impacts of public health actions. These unintended intervention-generated inequalities may operate through changing the risk across the other four pathways. For example, health communications which have not been culturally adapted to their target audiences may be less effective in some groups.^28^ Control measures that are inequitably implemented could mean that people in the same occupation experience different exposure risks. There is evidence to suggest that minority ethnic healthcare workers were less likely to be able to access personal protective equipment.^29^ In contrast, lockdown measures appeared to reduce COVID-19 mortality more among some minority ethnic groups than the majority population.^30^ The availability of effective vaccines is hugely welcome but could further exacerbate ethnic inequalities. For example, some minority ethnic groups (such as black and Pakistani/Bangladeshi ethnic groups) have higher levels of vaccine hesitancy in the UK.^31^ Importantly, differences in vaccine hesitancy reflect broader societal processes, such as the dominant communication strategies used in vaccination programmes and understandable concerns arising from longstanding experiences of discrimination (such as the Windrush scandal which led to the illegal deportation of black British citizens).

### Differential adverse consequences of control measures

Social and economic impacts of pandemic control measures (such as loss of income) may also disproportionately affect disadvantaged groups more and these impacts may affect non-COVID-19 health outcomes.^32^ Evidence already suggests that some minority ethnic groups have disproportionately experienced unemployment during the initial lockdown period and a greater increase in psychological distress, exacerbating existing ethnic inequalities in mental health.^33 34^

All of these six pathways arise from the wider social and political context that drive ethnic and other social inequalities, including structural racism and other power imbalances across society. Furthermore, multiple risks can affect multiple pathways—for example, poor working conditions might affect both the potential for differential exposure and experiencing differential consequences of control measures. Despite this, an understanding of the pathways driving ethnic inequalities can help identify policy targets.

## Conclusions

The COVID-19 pandemic has highlighted existing health inequalities among ethnic minority groups and exacerbated them. This has led to an increase in research studies to understand ethnic inequalities in health, but many research studies are based on a constrained and limited understanding of ethnicity and the potential pathways generating differences in health between ethnic groups. Ethnicity is a complex, multi-dimensional social construct and health differences between some ethnic groups largely reflect social pathways, embedded within the unequal power relationships that propagate inequalities. It is inappropriate for researchers to investigate ethnic groupings like biomarker or biomedical variables in naïve multivariable analysis that is not theoretically informed. In particular, overadjustment for mediating variables can lead to misleading interpretations, provide little insight to inform policy and practice and may ultimately have harmful real-world consequences.^35^ The unequal impacts of the pandemic can be mitigated, through more comprehensive and evidence informed action at each of the pathways we outlined above. This requires research that elucidates how specific dimensions of ethnicity differentially affect the mechanisms of differential exposure, vulnerability and consequences, identifying the most effective policy entry points to reduce ethnic inequalities in health. Our framework is a first step towards encouraging clearer thinking on ethnic inequalities in COVID-19 and we welcome feedback, anticipating that refinements will be needed over time.

**What is already known on this topic**

- Minority ethnic populations have experienced disproportionate harms during the COVID-19 pandemic.
- Considerable research is ongoing to understand the reasons for the greater risks being experienced, but a lack of theoretical underpinning for epidemiological analyses is often leading researchers to make erroneous conclusions.

**What this study adds**

- We present a framework for understanding the drivers of ethnic inequalities in COVID-19 harms, highlighting the multitude of mechanisms through which structural racism and power imbalances operate.
- Applying a theoretical framework can help policymakers and researchers develop more valid conclusions and ultimately better inform public health policies to mitigate adverse consequences of the pandemic on ethnic inequalities in health.

## Data Availability

Data sharing not applicable as no datasets generated and/or analysed for this study.
